# Emerging topics in FXTAS

**DOI:** 10.1186/1866-1955-6-31

**Published:** 2014-07-30

**Authors:** Deborah A Hall, Rachael C Birch, Mathieu Anheim, Aia E Jønch, Elizabeth Pintado, Joanne O’Keefe, Julian N Trollor, Glenn T Stebbins, Randi J Hagerman, Stanley Fahn, Elizabeth Berry-Kravis, Maureen A Leehey

**Affiliations:** 1 Department of Neurological Sciences, Rush University, Chicago, IL, USA; 2 Department of Developmental Disability Neuropsychiatry, School of Psychiatry, University of New South Wales, Sydney, Australia; 3 Département de Neurologie, Hôpitaux Universitaires de Strasbourg, Hôpital de Hautepierre, 67098 Strasbourg, Cedex, France; 4 Institut de Génétique et de Biologie Moléculaire et Cellulaire (IGBMC), INSERM-U964/CNRS-UMR7104/Université de Strasbourg, 67404 Illkirch, France; 5 Fédération de Médecine Translationnelle de Strasbourg (FMTS), Université de Strasbourg, Strasbourg, France; 6 Department of clinical Genetics, Kennedy Center, Rigshospitalet, Copenhagen University Hospital, Copenhagen, Denmark; 7 Department of Medical Biochemistry and Molecular Biology, University of Seville, Sevilla, Spain; 8 Department of Anatomy & Cell Biology, Rush University, Chicago, IL, USA; 9 Department of Pediatrics & M.I.N.D. Institute, University of California at Davis Medical Center, Sacramento, CA, USA; 10 Department of Neurology, Columbia University, New York, NY, USA; 11 Departments of Pediatrics and Biochemistry, Rush University, Chicago, IL, USA; 12 Department of Neurology, University of Colorado at Denver, Denver, CO, USA; 13 Centre for Healthy Brain Ageing, University of New South Wales, Sydney, Australia

**Keywords:** *FMR1*, FXTAS, Premutation, Fragile X, Tremor, Ataxia

## Abstract

This paper summarizes key emerging issues in fragile X-associated tremor/ataxia syndrome (FXTAS) as presented at the First International Conference on the *FMR1* Premutation: Basic Mechanisms & Clinical Involvement in 2013.

## Background

It has been over a decade since the fragile X-associated tremor/ataxia syndrome (FXTAS) was discovered. The clinical description of this new condition placed the disorder into the family of movement disorders. Movement disorders are neurological syndromes in which there is either an excess of movement (referred to as hyperkinesias, dyskinesia, or abnormal involuntary movements) or a paucity of voluntary and automatic movements (hypokinesia). Movement disorders are classified by their motor phenomenology. While many movement disorders have predominantly one type of abnormal movement, several diseases characteristically manifest a combination of abnormal movements. Because of the variety of motor phenomenology in FXTAS, patients with this condition are considered within the combinational movement disorders. In FXTAS, Parkinsonian and cerebellar features were initially described, but continued research suggests the disorder also includes non motor features, such as autonomic features, peripheral neuropathy, and neuropsychiatric manifestations.

The current definition of FXTAS is that it is caused by a premutation size expansion (55 to 199 CGG repeats) in the *fragile X mental retardation 1* (*FMR1*) gene. Full expansions of more than 200 CGG repeats in *FMR1* results in methylation and transcriptional silencing of the gene. Full mutation carriers have fragile X syndrome, which is characterized by intellectual disability, seizures, and autism with onset in childhood.

Unlike many other genetic disorders, the genetic abnormality that causes FXTAS was described and characterized before the discovery of the movement disorder. This has enhanced the ability of clinicians who identify children with fragile X syndrome (FXS) to rapidly identify parents and grandparents who may have FXTAS. It has also catalyzed the research in this disease. This paper is a summary of progress that has been made more recently in FXTAS and encompasses definitional changes, findings in women, classification of the cognitive disorders, measurement of clinical features, and report of data from the first clinical trial in the disorder.

## Expanding the FXTAS phenotype

The initial description of FXTAS consisted of a neurodegenerative disorder in premutation carriers, mostly in men over age 50, characterized by intention tremor, cerebellar gait ataxia, and parkinsonism, as well as brain atrophy and often middle cerebellar peduncle hyperintensities (the ‘MCP sign’) on magnetic resonance imaging (MRI) scans [1-4]. Diagnostic criteria [3], as shown in Table 1, were proposed based on this, with the addition of the neuropathological hallmark, intranuclear inclusion bodies [5], soon after. These diagnostic criteria have been helpful in clinical practice and research to identify affected persons [6,7].

**Table 1:** Fragile X-associated tremor/ataxia syndrome FXTAS diagnostic criteria

**FXTAS category:**^**a**^	**Exam and degree**	**Observation**
Definite**:**	Radiological	
One clinical major and one of the following:	*Major*	MRI white matter lesions in middle cerebellar peduncle and/or brain stem
• one radiological major criterion	Minor	MRI white matter lesions in cerebral white matter
• presence of intranuclear inclusion	Minor	Moderate to severe generalized atrophy
	Clinical	
Probable:	*Major*	Action tremor
One of the following:	*Major*	Cerebellar gait ataxia
	Minor	Parkinsonism
• two clinical major criteria	Minor	Moderate to severe short-term memory deficiency
• one radiological major criterion *and* one clinical minor criterion	Minor	Executive function deficit
	Pathological	
	*Major*	Classic FXTAS CNS intranuclear inclusions
Possible:		
Both of the following:		
• one clinical major criterion		
• one radiological minor criterion		

^a^For all categories, must have *FMR1* premutation. CNS, central nervous system; MRI, magnetic resonance imaging.

Since then there has been an enormous amount of literature suggesting that the disorder has additional features, as shown in Table 2. Further, some evidence suggests that REM sleep behavior disorder [8] and small fiber painful neuropathy [4] also occur in FXTAS. Thus, the original diagnostic criteria may be inadequate to best identify affected persons and need to be updated [4,9,10]. Indeed, given the accumulating literature, illness associated with the *FMR1* premutation includes a spectrum of disorders, depending on stage of life [10]. FXTAS, with core signs of action tremor and cerebellar gait ataxia, is a degenerative syndrome that occurs in late life and is the most severe end of this spectrum.

**Table 2:** Revised fragile X-associated tremor/ataxia syndrome (FXTAS) phenotype

**System affected**	**Symptoms and signs**	**Reference**
Motor		
	Action tremor	[1,3]
	Cerebellar gait ataxia	[1,3]
	Parkinsonism	[1,3]
	Reflex myoclonus	[11]
Cognitive		
	Executive dysfunction	[12]
	Dementia	[13]
Autonomic		
	Urinary dysfunction	[3]
	Erectile dysfunction	[3]
	Constipation/fecal incontinence	[12]
	Orthostatic hypotension	[14]
Psychiatric		
	Depression	[15-17]
	Anxiety	[15,17]
	Irritability, agitation, apathy	[16]
Other CNS, medical		
	Impaired olfaction	[8]
	Hearing loss	[8]
	Hypertension	[18,19]
	Sleep apnea	[20]
Peripheral nervous system		
	Length dependent neuropathy	[3]
	Non-length dependent sensory neuropathy	[4,8,21-28]
Females^a^		
	Muscle pain/fibromyalgia	[19,29]
	Hypothyroidism	[19]
Radiography		
	MRI T2 hyperintensities in the middle cerebellar peduncles (MCP sign) or brainstem	[4,30]
	MRI T2 hyperintensities in the splenium of the corpus callosum	[31]
	MRI white matter lesions in the cerebrum	[4,30]
	Moderate to severe generalized atrophy	[4,30]
	Corpus callosum atrophy	[4]
	Cerebellar atrophy	[4]

^a^Additional signs that are typical findings in females, much more so than males. CNS, central nervous system; MRI, magnetic resonance imaging.

Several additional clinical features may also occur in non-FXTAS carriers, do not presage the disease, and are not specific for FXTAS, such as chronic muscle pain, hypertension, and anxiety. However, these nonspecific signs often occur in early stages, and typical signs of FXTAS may be absent until more advanced stages. Of note, the expanded FXTAS clinical phenotype includes women, who tend to present differently than men as discussed in the next section.

**Figure 1:**
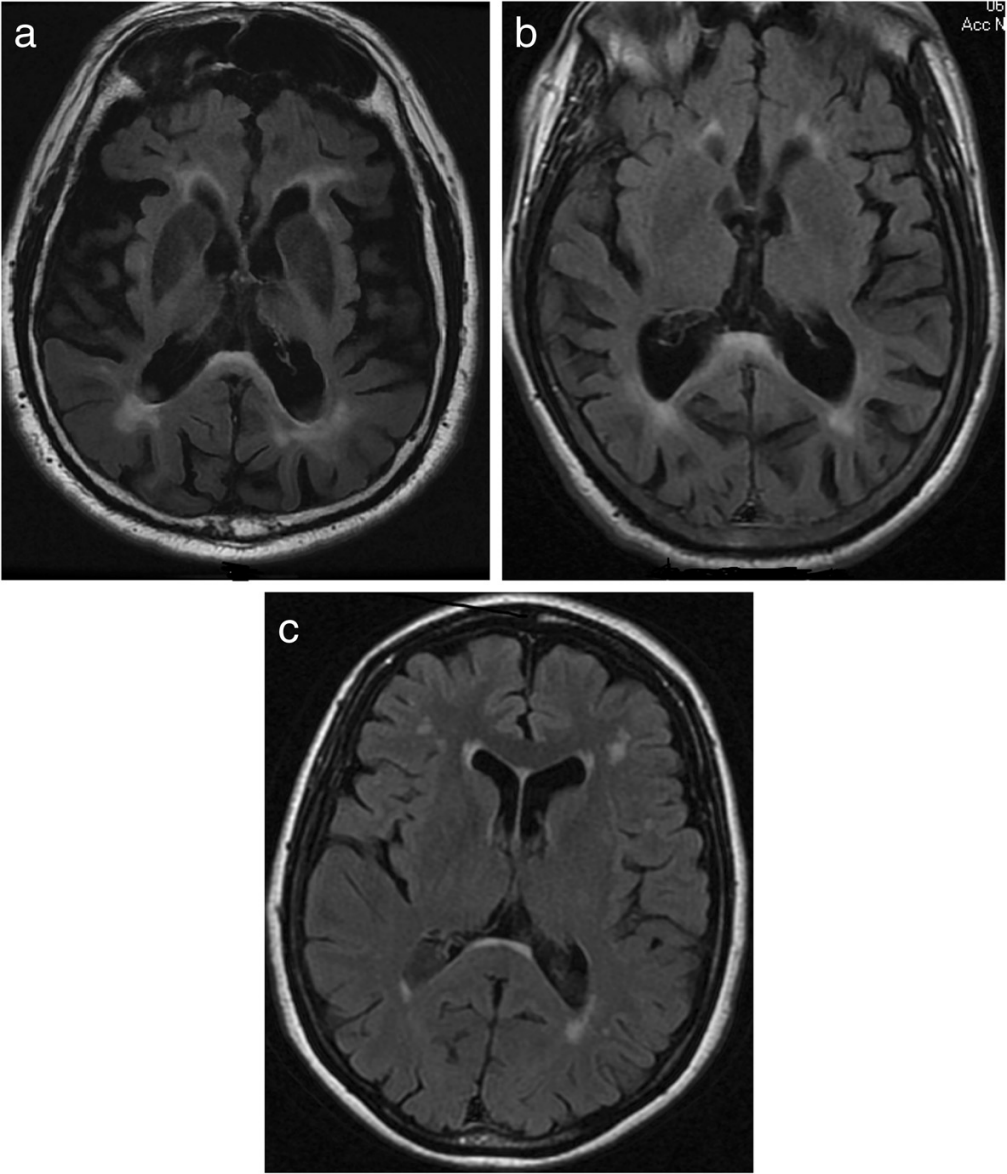
**Corpus callosum abnormalities in fragile X-associated tremor/ataxia syndrome (FXTAS).** Splenium of the corpus callosum hyperintensities on axial fluid attenuated inversion recovery (FLAIR) magnetic resonance images **(a, b and c)**.

Two major new findings in persons with FXTAS have been reported. A peripheral neuropathy has been known to be associated with FXTAS since the first cases. Apartis *et al.*[4], found that a non-length dependent neuropathy is found in 56% of premutation carriers with FXTAS. Thus, neuropathy is common enough to be a minor clinical diagnostic criterion, but too non-specific and common in the aging population to be classified as a major criterion. The same group [4] found that MRI T2 hyperintensities in the splenium of the corpus callosum splenium (CCS) were as frequent as MCP hyperintensities, and were useful in identifying patients who had no MCP sign (Figure 1). Therefore, CCS hyperintensities should be an additional major MRI criterion for the diagnosis of FXTAS.

FXTAS was initially described in *FMR1* premutation carriers. Recent reports, however, have shown that individuals carrying a gray zone [32,33] or full mutation without methylation [34] have developed a classic FXTAS picture, and thus would meet diagnostic criteria except that they were not premutation carriers. The pathologic mechanism that is proposed to underlie FXTAS is *FMR1* mRNA mediated neurotoxicity due to elevated levels of expanded repeat mRNA in the premutation range. Studies have shown that increased levels of mRNA levels begin in the gray zone [35,36]; this likely accounts for FXTAS occurring in individuals with these smaller alleles. Likewise, the occurrence of FXTAS in association with a full mutation was seen in a patient with an unmethylated allele [34]; and *FMR1* mRNA is significantly elevated in carriers of unmethylated full mutation alleles [37]. Given these findings, the diagnostic criteria for FXTAS need to be amended to allow for the diagnosis in individuals carrying gray zone or unmethylated full mutation alleles.

The current estimation of the prevalence of the tremor/ataxia phenotype in premutation carriers older than 50 years is 40% in men and 16% in women, recruited through families with known fragile X syndrome probands [19,38,39]. With recognition of the expanded phenotype and revision of the diagnostic criteria, identification of FXTAS will be increased. This allows earlier diagnosis and management of patients and their relatives, and is important because family members can be educated regarding their genetic and medical risks.

## FXTAS in women

The first carrier women of a *FMR1* premutation with FXTAS were reported in 2004 [6]. More women with FXTAS have since been described and FXTAS is now estimated to occur in 8 to 16% of premutation carrier women older than 50 years [6,19,39]. However, penetrance appears to be higher when there is a family history of FXTAS or other premutation problems such as immune mediated disorders [40,41]. As in men, the penetrance of FXTAS may increase with age in women, but this is less well documented [6]. Reduced penetrance in women is likely due to the protective effect of their normal allele on the second X-chromosome.

Women with FXTAS may be as severely affected as males, but in most women symptoms may vary in several respects from the original diagnostic criteria [42]. Women with FXTAS tend to have less tremor, ataxia, white matter disease and brain atrophy on MRI. In only 13% of females affected with FXTAS was the MCP sign observed [42]. In a small group of women affected with FXTAS (without a family history of FXS), the MCP sign was lacking in all patients and hyperintensities in the pons were less frequently observed [4]. In half of the women, hyperintensities of CCS were present. Some of the affected women presented with prominent parkinsonism [4].

Less dementia in late stage FXTAS has been reported in women [13,42], but a recent study suggests that dementia could be more common than initially described [43]. In this study, neuropathological findings in most premutation carrier women with dementia included cortical amyloid plaques and neurofibrillary tangles, making Alzheimer disease (AD) a possible cause of their dementia. The authors suggested that a synergistic effect could cause the disease progression in some FXTAS cases.

In contrast to premutation carrier men, medical co-morbidities are more common in women both with and without FXTAS. A higher incidence of hypothyroidism and fibromyalgia is reported in women with FXTAS than in men with FXTAS and age-matched controls [24,35]. Peripheral neuropathy, seizures and hypertension are also reported to be present more often in females with FXTAS than in controls [18,19]. Presence of FXTAS and other (neurological) disorders such as multiple sclerosis have been described in some carrier women [44,45]. Interestingly, although approximately 20% of carrier women have primary ovarian failure [46], one study reports that it is not associated with later onset of FXTAS [19].

## Emerging issues in clinical classification of cognitive disorders in FXTAS

Over 50% of patients with FXTAS show cognitive and behavioral changes, including impairment in executive function, processing speed and mood dysregulation, and increased risk for mood and anxiety disorders [8,13,15,16,31,47-49]. Additional features of apathy, disinhibition, and impairments in behavioral regulation suggest widespread dysfunction of the prefrontal lobe and its connections [12,16,50]. Collectively these features are consistent with the fronto-subcortical involvement that is commonly seen in association with other movement disorders including Parkinson disease (PD) and progressive supranuclear palsy [13,16].

Retrospective reports suggest that cognitive dysfunction emerges later than tremor and ataxia, [8,13] but many experience memory and executive dysfunction earlier than tremor or ataxia [50]. Cognitive dysfunction may be partly determined by disease duration or age. However, there is also evidence that cognitive dysfunction may be moderated by the *FMR1* gene. Larger CGG repeat expansions have been associated with increased relative risk for cognitive impairment [51] and specific impairments on measures of general intelligence [12,52,53], response inhibition and verbal fluency [54,55]. Moreover, volume loss and reduced activation in brain regions associated with working memory have been linked to larger CGG repeats and increased *FMR1* mRNA respectively [48,56].

The classification of the cognitive disorder associated with FXTAS has important research and clinical implications. A threshold for determining the presence of a cognitive disorder associated with FXTAS has not been established. The fifth edition of the Diagnostic and Statistical Manual (DSM-5) [57] introduces a new framework for classifying Mild or Major Neurocognitive Disorder (NCD) due to a variety of etiologies. This can be adopted for use in FXTAS. Cognitive impairment can be described as fulfilling criteria for Mild or Major NCD due to FXTAS, depending on the impact of cognitive symptoms on activities of daily living. Unlike its predecessor, DSM-5 does not require impairment in memory for a diagnosis of Mild or Major NCD, making the criteria more suitable for fronto-subcortical syndromes such as FXTAS.

The clinical classification of cognitive disorders in FXTAS can be challenging, especially in the elderly where multiple pathologies are more likely to co-exist. A number of post-mortem case studies examining brain tissue from patients with FXTAS have revealed evidence of co-morbid pathologies including multiple sclerosis, AD and dementia with Lewy bodies [44,45,58] in addition to FXTAS inclusions. Atypical cognitive phenotypes or atypical progression of symptoms may suggest the presence of co-morbid neurodegenerative conditions. Consideration of co-morbid degenerative syndromes has implications for clinical course and treatment. As postural instability and gait disturbance are associated with increased rates of cognitive decline in other motor disorders (for example, PD [59]), ataxia should be further evaluated as a possible association with cognitive decline in FXTAS. In fact, a study of 50 men with FXTAS suggested that cognitive impairment was positively associated with longer duration of ataxia [8].

## Measuring FXTAS

The FXTAS Rating Scale was created to capture the motor abnormalities, specifically tremor, ataxia and parkinsonism, in patients with FXTAS [52]. The scale was composed of items from the Clinical Rating Scale for Tremor [60], the International Cooperative Ataxia Rating Scale [61] and the Unified Parkinson’s Disease Rating Scale [62] with a tandem gait test added. The scale is designed to be administered by a neurologist trained to use the scale on either a live patient or a videotape of a patient, which captures the items to be rated. The items are all rated from a neurological exam and there is no patient-report or historical items. Clinimetric qualities of the scale have been evaluated by using scores from movement disorder neurologists blinded to gene status rating videotapes of premutation carriers using a structured videotape protocol. Four hundred and twenty-one individual ratings represented the gamut of FXTAS severity. Internal consistency, or the general agreement between items and the total score of the FXTAS Rating Scale was acceptable (Cronbach’s alpha = 0.93). However, some items had a less than ideal item to total score correlation, suggesting limited utility of these items for assessing overall FXTAS severity. The structure of the scale, when examined using exploratory factor analysis, encompasses ten domains or factors. These ten factors included measures of bradykinesia, ataxic signs, action tremor, rigidity, dysarthria, upper limb dystonia, rest tremor, lower limb dystonia, abnormal eye signs, and head tremor with dystonic voice. Although these ten factors were identified, many of the individual items were associated with multiple factors, suggesting a lack of domain specificity for those items. To determine the responsivity of the scale, retrospectively collected FXTAS Rating Scale scores of premutation carriers between 2001 and 2012 were utilized. Mean follow up time of the subjects (n = 67) was 38.5 months and mean change in score from baseline to follow up was +4.32 points (SD = 13.46). This was calculated to be an average yearly change of +2.55 points (95% CI was −12.44 to +17.53). The studies show that FXTAS Rating Scale has good internal consistency, but specific items need to be altered or eliminated to complete development of the scale.

Falls and balance issues are clearly a major aspect of FXTAS and the FXTAS Motor Rating Scale may not be sensitive enough to detect early balance dysfunction in the disease. Highly sensitive early markers of disease onset are critically needed to characterize the subtle balance deficits that may not be identifiable on the neurological exam in early FXTAS or in premutation carriers without FXTAS. Computerized Dynamic Posturography (CDP) with a Neurocom® Balance Master has been used in a small series of premutation carriers with and without a definitive diagnosis of FXTAS [63] to investigate balance in this population compared with normative data from a group of healthy controls provided by the Neurocom® manufacturer.

**Figure 2:**
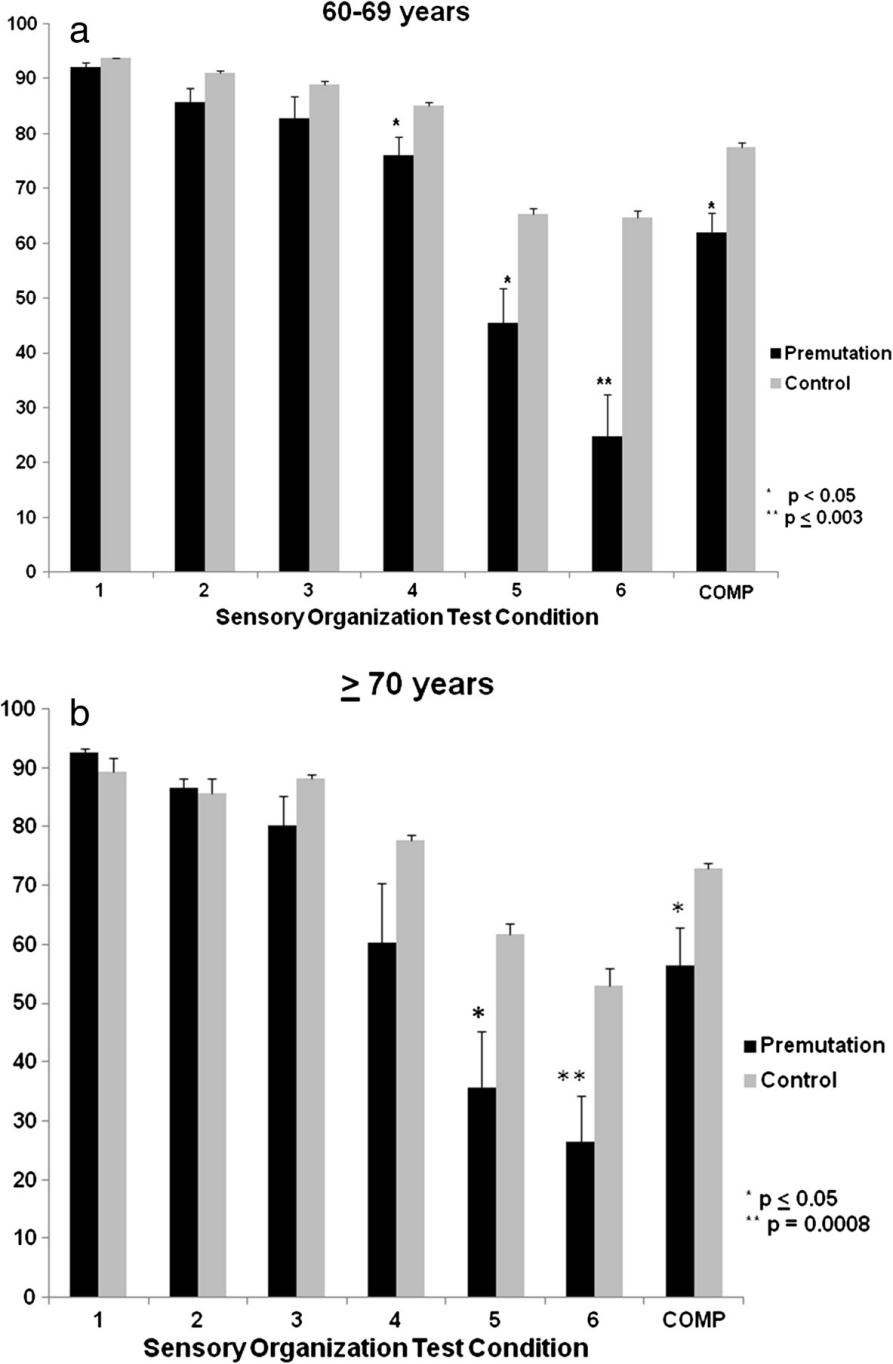
**Computed dynamic posturography in fragile X-associated tremor/ataxia syndrome (FXTAS). (a and b)** Neurocom® Computerized Dynamic Posturography (CDP) Equilibrium scores on all six conditions of the Sensory Organization Test (SOT) and the SOT composite (COMP) score between *FMR1* premutation carriers and controls in the 60 to 69 year and the ≥ 70 year-old age group. Data is expressed as mean ± SEM. Lower SOT scores signify greater postural sway. Worse performance on conditions 5 and 6 of the SOT reflect the inability to appropriately use vestibular information for the control of balance.

CDP testing, in premutation carriers over age sixty (n = 8 between 60 and 69 years of age and n = 6 greater than 70 years of age), demonstrated abnormally low scores on conditions of the Sensory Organization Test (SOT) that reflect vestibular system balance deficits (See Figure 2; *P* = 0.02 to < 0.001). In the SOT, the relative somatosensory, visual and vestibular contributions to balance control are tested by evaluating balance in six different conditions in which somatosensory and/or visual inputs in various combinations are eliminated or made unreliable for maintenance of balance. On the CDP motor control test, which measures latency of automatic postural responses to balance disruption, response latencies to medium and large forward and backward translations were significantly longer in older premutation carriers (0.002 > *P* < 0.0001). The CDP limits of stability test, which measures maximum distance one can displace their center of gravity without stepping or falling, showed significantly reduction for maximum excursion to targets and directional control while moving toward a target, in the premutation carrier group at all ages tested (0.05 ≥ *P* < 0.0001). Scores on vestibular conditions of the SOT were reduced not only in carriers with evidence of FXTAS on exam and on the FXTAS Rating Scale, but also in a subset of carriers with normal neurological exams and normal scores on the FXTAS Rating Scale. A ‘Balance subscale’ score of the FXTAS Rating Scale scores correlated with severity of the deficit on vestibular SOT conditions (r = 0.56; *P* = 0.02). These analyses suggest that CDP may be a more sensitive balance ‘marker’ than the FXTAS Rating Scale in detecting preclinical disease and for risk prediction. Larger longitudinal studies are needed to fully validate and develop this and other such measures.

## Memantine for FXTAS

Memantine, an antagonist of *N*-methyl-D-aspartate (NMDA) glutamate receptor approved for the treatment of moderate to severe AD, has been suggested to be of therapeutic benefit in a number of neurological disorders associated with over-activation of glutamate receptors, including AD, PD, and Huntington’s disease [64]. Anecdotal reports have suggested that memantine could be helpful in the treatment of FXTAS [65]. There is evidence of down-regulation of GABA pathways causing [66] excessive glutamate to remain at the synapse leading to possible glutamate toxicity [24]. Based on this mechanism, a randomized, double-blind, placebo-controlled trial of memantine in individuals with FXTAS ages 34 to 80 years was carried out [25]. Ninety-four participants were randomized to either memantine or placebo and were treated for one year. There was no improvement over placebo in the primary outcome measures of intention tremor severity as measured by the CATSYS (memantine versus placebo: 1.01 ± 0.75 versus 1.76 ± 2.13, *P* = 0.0785) or executive function as measured by the Behavior Dyscontrol Scale (16.12 ± 5.43 versus 15.72 ± 3.93, *P* = 0.7268) in an intention-to-treat analysis. More mild adverse events (AEs) were observed in the placebo group and more moderate AEs occurred in the memantine group (*P* = 0.007). This study suggests that memantine is not helpful for the primary problems of FXTAS, specifically tremor and executive function.

A sub-study using event related potentials (ERP) in a word repetition paradigm was performed in order to elicit N400 repetition effects with congruous and incongruous words. There were 21 patients in the memantine group and 20 in the placebo group who underwent successful ERP studies before and after one year of treatment. The memantine group exhibited larger improvements on the cued recall test for targeted words compared to the placebo group (*P* = 0.050). The placebo group displayed statistically significant reduction of the N400 repetition effect after one year, while the treated group showed preservation of the N400 repetition effect, with a significant trend for larger N400 repetition effect amplitude after one year of treatment with memantine [26]. Correlational tests revealed that increased N400 repetition effect amplitudes were associated with improved cued-recall scores for the congruous target words (one year to baseline) across all subjects (*r* = 0.36, *P* = 0.02), and in the memantine group (*r* = 0.46, *P* = 0.038), but not within the placebo group (*r* = 0.17, *P* = 0.46). This sub-study suggests that the ERP paradigm may be a sensitive measure to document some limited benefit from memantine in individuals with FXTAS.

## Future directions

While the prevalence of the FXTAS is estimated to be similar to that of other neurodegenerative disorders [27], FXTAS is under-recognized and frequently misdiagnosed [28]. While neurologists are becoming more aware of FXTAS, approximately half of affected persons are seen and managed by primary care physicians [28]. Improved education of physicians and more accurate, broader diagnostic criteria, as in Table 3, would improve diagnosis. Health care professionals especially need to be educated about the expanded female phenotype, as these carriers have serious genetic and medical risks. Keeping in mind that a family history should be checked, but also that the family history may be negative for clear *FMR1* related disorders, health care professionals should consider gene testing if a patient over age 50 has unexplained cerebellar gait ataxia, unexplained action tremor and dementia, or the MCP or CCS hyperintensity on MRI and some FXTAS signs, as in Table 2.

**Table 3:** Revised fragile X-associated tremor/ataxia syndrome (FXTAS) diagnostic criteria

**FXTAS category:**^**a**^	**Exam and degree**	**Observation**
Definite:	Radiological	
One clinical major and one of the following:	*Major*	MRI white matter lesions in middle cerebellar peduncle
• one radiological major criterion	*Major*	MRI white matter lesions in splenium of the corpus callosum
• presence of intranuclear inclusion	Minor	MRI white matter lesions in cerebral white matter
	Minor	Moderate to severe generalized atrophy
	Clinical	
Probable:	*Major*	Action tremor
One of the following:	*Major*	Cerebellar gait ataxia
• two clinical major criteria	Minor	Parkinsonism
• one radiological major criterion *and* one clinical minor criterion	Minor	Moderate to severe short-term memory deficiency
	Minor	Executive function deficit
	Minor	Neuropathy
	Pathological	
	*Major*	Classic FXTAS CNS intranuclear inclusions
Possible:		
Both of the following:		
• one clinical major criterion		
• one radiological minor criterion		

^a^For all categories, must have *FMR1* gray zone, premutation or full mutation.

Many important avenues of research need attention. The female FXTAS phenotype requires further study to accurately define medical risks, especially regarding endocrine dysfunction and chronic pain. Genetic, epigenetic and environmental factors that predispose carriers to develop FXTAS need identification. Further, to date only one randomized, controlled trial to study modification of progression of FXTAS has been conducted. The tools for these studies, for example, the FXTAS Rating Scale and other motor measures such as the Neurocom®, need refinement, and the molecular science behind such trials needs to be established.

## Abbreviations

CDP: Computerized dynamic posturography; CNS: Central nervous system; ERP: Event related potential; *FMR1*: *Fragile X mental retardation 1* gene; FXS: Fragile X syndrome; FXTAS: Fragile X-associated tremor/ataxia syndrome; MCP: Middle Cerebellar Peduncle; MRI: Magnetic resonance imaging; NCD: Neurocognitive disorder; REM: Rapid eye movement.

## Competing interests

EBK: EBK has received clinical trial funding and consulting fees from Novartis, Roche and Seaside Therapeutics and has received funding to develop standards for *FMR1* testing from Asuragen, Inc. She is also on advisory boards for Genentech, Roche and Novartis regarding treatment of FXS.

RJH has received funding from Novartis, Roche, Seaside Therapeutics, Forest and Curemark for clinical trials in FXS or autism. She is also on advisory boards for Genentech, Roche and Novartis regarding treatment of FXS. The Kennedy Center, Denmark has received clinical trial funding from Novartis. All the other authors have no competing interest to declare.

## Authors’ contributions

DH: manuscript planning, drafting of initial manuscript, final manuscript editing and submission. EBK: drafting of initial manuscript, final manuscript editing. RH: drafting of initial and final manuscript editing. RB: drafting of initial manuscript, final manuscript editing. JNT: drafting of initial manuscript, final manuscript editing. ML: manuscript planning, drafting of initial manuscript, final manuscript editing. MA: drafting of the manuscript, critical revision of the manuscript. AEJ: drafting of initial manuscript, final manuscript editing. EP: drafting of initial manuscript, final manuscript editing. GS: final manuscript editing. All authors read and approved the final manuscript.
