# Erratum: Emerging topics in FXTAS

**DOI:** 10.1186/s11689-015-9108-7

**Published:** 2015-04-08

**Authors:** Deborah A Hall, Rachael C Birch, Mathieu Anheim, Aia E Jønch, Elizabeth Pintado, Joan A O’Keefe, Julian N Trollor, Glenn T Stebbins, Randi J Hagerman, Stanley Fahn, Elizabeth Berry-Kravis, Maureen A Leehey

**Affiliations:** Department of Neurological Sciences, Rush University, Chicago, IL USA; Department of Developmental Disability Neuropsychiatry, School of Psychiatry, University of New South Wales, Sydney, Australia; Département de Neurologie, Hôpitaux Universitaires de Strasbourg, Hôpital de Hautepierre, Strasbourg, Cedex 67098 France; Institut de Génétique et de Biologie Moléculaire et Cellulaire (IGBMC), INSERM-U964/CNRS-UMR7104/Université de Strasbourg, Illkirch, 67404 France; Fédération de Médecine Translationnelle de Strasbourg (FMTS), Université de Strasbourg, Strasbourg, France; Department of clinical Genetics, Kennedy Center, Rigshospitalet, Copenhagen University Hospital, Copenhagen, Denmark; Department of Medical Biochemistry and Molecular Biology, University of Seville, Sevilla, Spain; Department of Anatomy & Cell Biology, Rush University, Chicago, IL USA; Department of Pediatrics & M.I.N.D. Institute, University of California at Davis Medical Center, Sacramento, CA USA; Department of Neurology, Columbia University, New York, NY USA; Departments of Pediatrics and Biochemistry, Rush University, Chicago, IL USA; Department of Neurology, University of Colorado at Denver, Denver, CO USA; Centre for Healthy Brain Ageing, University of New South Wales, Sydney, Australia

## Erratum

Following the publication of our article [1], we noticed that Dr Joan A O’Keefe and Dr Stanley Fahn had inadvertently been omitted from the authors’ contributions section. In addition, Dr Joan A O’Keefe was incorrectly listed in the author list as Joanne O’Keefe. The author list has now been corrected and the amended authors’ contributions section is included below.
